# KDM3A Inhibition Ameliorates Hyperglycemia-Mediated Myocardial Injury by Epigenetic Modulation of Nuclear Factor Kappa-B/P65

**DOI:** 10.3389/fcvm.2022.870999

**Published:** 2022-04-29

**Authors:** Bofang Zhang, Jing Zhang, Gen Liu, Xin Guo, Xiaopei Liu, Jing Chen

**Affiliations:** ^1^Department of Cardiology, Renmin Hospital of Wuhan University, Hubei Key Laboratory of Cardiology, Cardiovascular Research Institute, Wuhan University, Wuhan, China; ^2^Department of Cardiology, The First College of Clinical Medical Science, Yichang Central People’s Hospital, China Three Gorges University, Yichang, China

**Keywords:** hyperglycemia, cardiac dysfunction, epigenetic regulation, KDM3A, NF-κB/p65

## Abstract

**Objectives:**

Even after the glucose level returns to normal, hyperglycemia-induced cardiac dysfunction as well as reactive oxygen species (ROS) generation, inflammatory responses, and apoptosis continued deterioration, showing a long-lasting adverse effect on cardiac function and structure. We aimed to unveil the molecular and cellular mechanisms underlying hyperglycemia-induced persistent myocardial injury and cardiac dysfunction.

**Methods and Results:**

Recently, the accumulated evidence indicated epigenetic regulation act as a determining factor in hyperglycemia-induced continuous cardiovascular dysfunction. As an important histone demethylase, the expression of lysine-specific demethylase 3A (KDM3A) was continually increased, accompanied by a sustained decline of H3K9me2 levels in diabetic myocardium even if received hypoglycemic therapy. Besides, by utilizing gain- and loss-of-functional approaches, we identified KDM3A as a novel regulator that accelerates hyperglycemia-mediated myocardial injury by promoting ROS generation, aggregating inflammatory reaction, and facilitating cell apoptosis *in vitro* and *in vivo*. The KDM3A inhibition could significantly ameliorate the adverse effect of hyperglycemia in both diabetes model and diabetic intensive glycemic control model. Mechanically, our data uncovered that KDM3A could promote the expression and transcriptional activity of nuclear factor kappa-B (NF-κB/P65), and the succedent rescue experiments further verified that KDM3A regulates hyperglycemia-induced myocardial injury in an NF-κB/P65 dependent manner.

**Conclusion:**

This study revealed histone-modifying enzymes KDM3A drives persistent oxidative stress, inflammation, apoptosis, and subsequent myocardial injury in the diabetic heart by regulating the transcription of NF-κB/P65.

## Introduction

As an important systemic metabolic disorder, diabetes mellitus (DM) has become one of the most challenging and prevalent public health issues worldwide over the last few decades (1, 2). Diabetes mellitus emerges as a major risk factor for the development of cardiovascular disease, which eventually serves as the principal cause of morbidity and mortality among the diabetic population (3, 4). In addition, DM induces specific cardiac functional and structural abnormalities leading to diabetic cardiomyopathy (DCM), which is characterized by myocardial hypertrophy, fibrosis, and ventricular dysfunction that occur independently of hypertension and ischemic coronary artery disease (5, 6). Although the pathophysiology of DCM is complex and multifactorial, hyperglycemia, as the primary clinical manifestation of DM, is considered to be the main etiological factor of myocardial damage in this setting (7, 8). An increasing body of evidence suggests that hyperglycemia can trigger a series of pathological molecular pathways, leading to overloaded accumulation of reactive oxygen species (ROS), exaggerated cardiac inflammatory responses, and excessive cytokines release (9, 10). Meanwhile, intense oxidative stress and inflammation will crosstalk and reinforce with each other, ultimately inducing apoptosis of cardiomyocytes (11, 12). Oxidative stress, inflammation, and apoptosis were engaged throughout the pathological process of hyperglycemic myocardial injury (13).

Theoretically, given the deleterious effects of high glucose (HG) on myocardial tissue, intensive glycemic control (GC) therapy was believed as an effective way to attenuate myocardial damage caused by hyperglycemia at the very beginning. Unexpectedly, evidence is building up from recent subclinical experiments and large randomized trials that even optimal GC failed to reduce cardiovascular complications and outcomes (14). The hyperglycemia stress seems to be remembered in diabetic heart and provoke sustained myocardial damage, even though blood glucose level returned to normal. Meanwhile, the persistent activation of oxidative stress, inflammation, and apoptosis were also observed in this pathological state (15, 16). Fortunately, the conclusions from several lines of experiments have shed the light on the solution to the tough issue. For example, it has been uncovered that only those patients with diabetics who received a combination of insulin and vitamin C at high doses (antioxidant stress) could efficiently reverse the detrimental effects of hyperglycemia (17). As the multifaceted mechanisms of glucotoxicity include long-lasting oxidative stress, cardiac inflammatory responses, and cardiomyocyte apoptosis, a therapeutic approach combatting both these underlying mechanisms and hyperglycemia could be a promising strategy to prevent progressive myocardial damage.

Epigenetics is characterized by heritable changes in gene activity and expression without altering the DNA sequence, thus exerting a long-lasting regulatory effect in gene expression (18, 19). Epigenetic changes, especially histone modification have been postulated and considered as key determinants of hyperglycemia-induced continuous cardiovascular dysfunction only recently (20, 21). It has been demonstrated that vascular smooth cells (VSMCs) from diabetic mice exhibit higher expression of inflammatory genes, associated with lower levels of the histone lysine methyltransferases SUV39H1 and lower levels of promoter H3K9me3 (22). Besides, SIRT1-dependent deacetylation of p66*^Shc^* promoter persistently existed in HG treated cardiomyocytes and was not altered by intensive GC, resulting in excessive ROS generation and cellular death (15). The lysine-specific demethylase 3A (KDM3A, also named JMJD1A) is a well-known epigenetic activator heralding target gene transcription *via* the removal of suppressive histone mark di-methylation of histone H3 at lysine 9 (H3K9me2) (23). Numerous studies have confirmed its pathophysiological significance in cardiovascular disease (24, 25). Besides, our prior study has affirmed that KDM3A-mediated H3K9me2 erosion plays a crucial role in diabetic vascular remodeling (26). In addition, the benefits deriving from KDM3A inhibition on high insulin-induced VSMCs malfunctions also have been shown, manifesting as a therapeutic approach due to pleiotropic potencies in the regulation of and ROS, inflammation, and apoptosis (27). Thus, KDM3A was identified as a pivotal regulator in both epigenetic modification and ROS, inflammation, and apoptosis-associated signaling pathways. In the light of these considerations, we speculated that KDM3A may also play a central role in the regulatory network of hyperglycemia-induced sustained myocardial damage.

This study was designed to determine whether KDM3A was involved in modulating hyperglycemia-induced continuous myocardial injury as well as to explore the underlying molecular biological mechanisms. By establishing hyperglycemia and subsequently intensive GC myocardial injury models both *in vitro* and *in vivo*, our experiment provides compelling evidence that KDM3A inhibition could epigenetically modification of NF-K b/P65 by increasing the level of H3K9me2, which consequently ameliorate hyperglycemia-induced persistent myocardial damage and cardiac dysfunction.

## Materials and Methods

### Animals

The Specific Pathogen Free (SPF)-grade male wild type (WT) SD rats (200–250 g) and neonatal rats (1–3 days) were provided by Wuhan University Experiment Animal Center. The rats were hosted in an environment with 50–60% humidity and a 12 h:12 h light–dark cycle at 22–24°C, and with free access to food and water. The animal experiments were performed in strict accordance with the published NIH Guidelines for the Care and Use of Laboratory Animals and approved by the Animal Care and Use Committee of Wuhan University.

### Construction of KDM3A Knock-Out Rats

A CRISPR/Cas9 genome-editing technology was operated to generate KDM3A global knock-out rats (KDM3A-KO rat, SD background) as described in our previous study (25). Briefly, one single guide RNA (sgRNA) flanked exon 5 of the KDM3A gene in rat was designed and created. When purified Cas9 mRNA and sgRNA were mixed and microinjected into embryos, PCR products were TA cloned and sequenced to define the exacted indel mutations of generated founders. The primers were listed as follows: KDM3A F: 5′-TCCCTGGAGTTGCAGTAGTTT-3′; KDM3A R: 5′-AGGTCTATCACTGGCTAACTCA-3′. The founder rat was chosen as the breeding line and mated with the WT (kdm3a^+/+^) SD rat strain to generate F1 heterozygote (kdm3a^±^). The homozygous kdm3a^–/–^ strain was obtained through sibling mating of heterozygous F1 offspring and was identified by sequencing chromatograms of PCR products. The western blotting assay was also performed to validate KDM3A protein expression.

### Establishment of Hyperglycemia Myocardial Damage Model and Intensive Glycemic Control Model *in vivo*

The rats received a high-fat diet for 4 weeks and were intraperitoneally injected with a low dose of streptozocin (STZ) (30 mg/kg) to establish a reliable type 2 diabetes mellitus (T2DM) model (fasting blood glucose >13.9 mmol/L). Subsequently, these T2DM rats were fed with a high-fat diet for another 16 weeks and received subcutaneous injections of saline daily from the time point of 8 weeks to construct an *in vivo* hyperglycemia myocardial damage model (DM group). Meanwhile, T2DM rats were fed with a high-fat diet for another 16 weeks and received subcutaneous injections of insulin (Novolin N, 3 U/d) from the time point of 8 weeks to establish an *in vivo* intensive GC model (DM–GC). The rats in the Sham group were fed with a normal diet for 20 consecutive weeks.

### Culture and Treatment of Primary Cardiomyocytes

The primary cardiomyocytes were isolated from the heart of 1–3-day-old rats by differential adhesion method and identified by morphology and immunofluorescence as previously described (25). Subsequently, the cells in the normal glucose (NG) group were incubated with Dulbecco’s modified Eagle’s medium (DMEM)/F12 containing 5.5-mM glucose for 72 h. Cells in the HG group were treated with DMEM/F12 containing 30-mM glucose for 72 h to establish an *in vitro* hyperglycemia myocardial injury model. Cardiomyocytes in the HG–NG group were cultivated with a medium containing 30-mM glucose for 24 h followed by incubated with a medium containing 5.5-mM glucose for 48 h to establish a diabetic intensive GC model *in vitro*.

### Transfection of Adenovirus Vectors and Plasmid

The short-hairpin RNA adenoviruses AdshKDM3A (KDM3A inhibition) and its negative control AdshRNA, as well as AdKDM3A (KDM3A over-expression) and its negative control AdGFP, were constructed by Shanghai GeneChem Co., Ltd. The primary cardiomyocytes were seeded into a 6-well plate at 10^5^/well and infected with various multiplicities of infection (MOI). The cells were transiently transfected for 2 h and continuously transfected for 48 h after the original medium was replaced by the complete medium. Finally, the number of GFP-positive cells and their activities were observed under a fluorescence microscope, and the cells with an optimal MOI value were selected for the subsequent experiments.

The siNF-κB/P65 plasmid was synthesized by RiboBio (Guangzhoug, China) and transfected with Lipofectamine 2000. The primary cardiomyocytes were transfected with siNF-κB/P65 plasmid for 6 h followed by incubated with normal medium for another 48 h.

### Detection of Oxidative Stress

At the cellular level, the primary cardiomyocytes were incubated at 37°C for 30 min with a dihydroethidium (DHE) fluorescent probe (Beyotime Institute of Biotechnology Co., Ltd., China), at a final concentration of 10 μmol/L. Under the fluorescence microscope, ROS-positive cells were dyed in red in the whole nuclear region. Later, at least five fields of view were randomly selected, photographed, and analyzed by the ImageJ software. Besides, a flow cytometry assay (FACS Calibur; BD Biosciences, United States) was also used to analyze the percentage of DHE positive cells. To further detect the intercellular superoxide generation and lipid peroxidation, the malondialdehyde (MDA) concentration and the superoxide dismutase (SOD) enzymatic activity in cardiomyocytes were also measured, in accordance with the descriptions of commercial kits (Nanjing Jiancheng Bioengineering Institute, China). To evaluate ROS generation in myocardial tissue, fresh frozen left ventricular samples (5-μm sections) were incubated with DHE (2 mmol/L) for 1 h at room temperature in the dark. The slides were visualized by fluorescence microscopy, and the mean fluorescence intensity (MFI) of each slide was quantified by Image-Pro Plus software version 6.0. The MDA concentration and SOD enzymatic activity in fresh myocardial tissue homogenate were also measured in the same manner as that *in vitro*.

### Quantification of Apoptotic Cells by Flow Cytometry *in vitro*

Flow cytometry was used to estimate apoptotic cells as previous demonstrations (27). The cardiomyocytes in different groups were harvested and resuspended in 1 × binding puffer at a final concentration of 1 × 10^6^ cells/ml. Then, 5 μl of 7-aminoactinomycin D (7-AAD) was added into the cell suspension and then the cells underwent further staining with Annexin V-APC (AV-APC) dye, followed by the fluorescence-activated cell sorting on a flow cytometric assay (CytoFLEX; Beckman Coulter, Inc., Brea, CA, United States). The Annexin V-APC-positive cells were identified as early apoptosis, while both annexin V-APC- and 7-AAD-positive cells were identified as late apoptosis. The total apoptosis rate comprised the proportions of early apoptotic cells and late apoptotic cells.

### Measurement of Inflammation

The expression of inflammatory cytokine was detected by commercialized enzyme-linked immunosorbent assay (ELISA) kits (Nanjing Jiancheng Bioengineering Institute, Nanjing, China). The cardiomyocytes and fresh myocardial tissues were collected and crushed into homogenate to examine the levels of pro-inflammatory mediator tumor necrosis factor-alpha (TNF-α) and Interleukin-6 (IL-6) according to the manufacturer’s instructions.

### Histological Examination

The myocardial tissues were fixed with 4% paraformaldehyde for 72 h, dehydrated, transparentized, and embedded in paraffin to prepare 4-μm slices. The paraffin sections were stained with an *in situ* TUNEL detection kit (Roche, Basel, Switzerland) to detect apoptotic cells as previously described (28). Red fluorescent dye and 4′,6-Diamidino-2′-phenylindole (DAPI) were performed to specifically mark apoptotic nuclei and overall nuclei, respectively. The TUNEL-positive nuclei (red) and total cells (blue) were photographed with a fluorescent microscope (Olympus, Tokyo, Japan) in at least five randomly selected fields per section (200 × magnification). The apoptotic index was expressed as the ratio of apoptotic cells over total myocytes of DAPI-dyed nuclei. Hyperglycemia-induced myocardial remodeling after infarction was determined by Masson’s staining, areas stained in blue indicate the deposition of collagen fibrils. Hematoxylin and eosin (H&E) and wheat germ agglutinin (WGA) staining were also performed to detect cardiac hypertrophy as previously described (25).

### Assessment of Cardiac Function

Transthoracic echocardiography was carried out to determine cardiac function after animals were lightly anesthetized by inhaling 1.5–2.5% isoflurane. The transthoracic echocardiograms were recorded using MyLab 30CV ultrasound system (Biosound Easote, Inc.). Two-dimensional (2D) images were collected in the short axes with at least three representative cycles. Left ventricular (LV) M-mode tracing at mid-papillary levels was measured and averaged for the calculation of ejection fraction (EF) and factional shortening (FS).

### Dual-Luciferase Reporter Gene Assay

The primary cardiomyocytes were transfected with AdKDM3A or AdshKDM3A. The pGL6-nuclear factor kappa-B (NF-κB) plasmid DNA, pRL-TK plasmid DNA (Beyotime, Shanghai), and Lipofect (Ribobio, Guangzhou) were prepared according to the manufacturer’s instructions and were used to transfect the cells. Subsequently, the cells were fully lysed and centrifuged to obtain the supernatant. Thereafter, 100 μl of Firefly luciferase detection reagents (Beyotime, Shanghai) was added to 50 μl of samples and the relative light unit (RLU) was determined on the machine. The reporter gene cell lysate was used as a blank control. Thereafter, 100 ml of Renilla luciferase working solution (Beyotime, Shanghai) was added to continuously measure the RLU and the ratio of Firefly luciferase/Renilla luciferase was calculated in each tube.

### Electrophoretic Mobility Shift Assay

The proteins were extracted from the nucleus, and their concentration was determined by the bicinchoninic acid (BCA) assay (ASPEN). An electrophoresis gel was prepared according to the manufacturer’s instructions (Thermo Scientific), and the proteins were subjected to pre-electrophoresis and electrophoresis and transferred onto a membrane, followed by cross-linking for 10 min. After 15 min of blocking, the membrane was incubated with a diluted antibody for 15–20 min, washed with the eluent and equilibrated for 5 min using the equilibration solution. Finally, enhanced chemiluminescence (ECL) was used for image development, and the AlphaEaseFC software processing system was used to analyze the bands.

### Western Blotting Assay

The Radio Immunoprecipitation Assay (RIPA) lysis buffer (Roche) was used to extract total proteins from the heart tissues or cells. After quantitative analysis by BCA, the samples were separated by 8–12% SDS–PAGE and then transferred onto PVDF membranes (Merck Millipore). After blocked by 5% skim milk, the membranes were incubated with corresponding primary (KDM3A ab106456, H3K9me2 ab1220, NF-κB/P65 ab194726) and secondary antibodies at the recommended dilution. The glyceraldehyde-3-phosphate dehydrogenase (GAPDH) (ab37168) was chosen as an internal control. The protein bands were visualized using an ECL system (Thermos Fisher Scientific, Inc).

### Statistical Analysis

All data were analyzed using the SPSS 19.0 software package. The measured data were expressed as mean ± standard deviation. Student’s *t*-test was used to compare between two groups, and one-way analysis of variance (ANOVA) was applied for the comparison among multiple groups. The value, *p* < 0.05, represented a statistically significant difference. All results were independently reproduced at least 3 times with similar results.

## Results

### High Glucose-Induced Cardiomyocyte Injury Persisted Even After Glucose Levels Were Normalized, Accompanied by the Over-Expression of KDM3A

**FIGURE 1:**
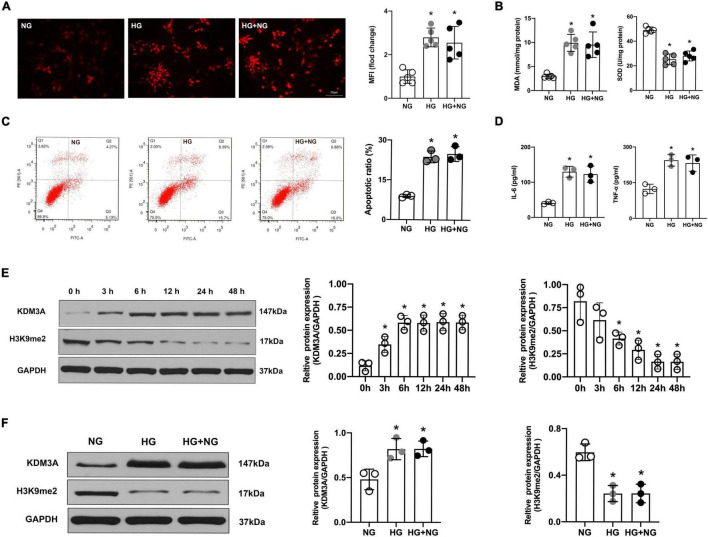
High glucose-induced cardiomyocyte dysfunction persist even after glucose levels were normalized, accompanied by the over-expression of KDM3A. **(A)** ROS generation was detected by the DHE method, and the MFI was a quantitative indicator for analysis (*n* = 5, *p < 0.05, as compared to the NG group). **(B)** The MDA content and SOD activity of cell lysate were assessed simultaneously (*n* = 5, *p < 0.05, as compared to the NG group). **(C)** The apoptosis of cardiomyocytes in different groups was tested by flow cytometry (*n* = 3, *p < 0.05, as compared to the NG group). **(D)** The inflammatory factors IL-6 and TNF-α in cardiomyocytes of each group were measured by ELISA kits (*n* = 3, *p < 0.05, as compared to the NG group). **(E)** At the cellular level, the protein expression of KDM3A and H3K9me2 were detected with HG treatment from 0 to 48 h (*n* = 3, *p < 0.05, as compared to the 0 h group). **(F)** The protein levels of KDM3A and H3K9me2 in cardiomyocytes of NG, HG, and HG–NG the group were measured by western blot (*n* = 3, *p < 0.05, as compared to the NG group).

According to the results of DHE staining, HG treatment results in evident accumulation of ROS in the cardiomyocyte. However, there was no significant difference in the MFI between the HG and the HG + NG groups (Figure 1A). Besides, as shown in Figure 1B, the HG obviously improved the level of MDA but decreased the level of SOD. Moreover, HG-induced oxidative stress continued to be activated even though the HG medium was replaced by an NG medium (Figures 1A,B). In addition, the results of flow cytometry and ELISA also showed that HG treatment remarkably facilitated the apoptotic ratio of cardiomyocytes as well as markedly increased expression of the IL-6 and TNF-α. However, the restoration of NG neither obviously reduce the apoptosis of cardiomyocytes nor distinctly suppress the expressions of inflammatory factors (Figures 1C,D). The above results suggest that HG treatment could induce continuous cardiomyocytes dysfunction, and the sustained activation of ROS, apoptosis and inflammatory responses might be the vital underlying pathophysiological mechanism.

Interestingly, as a key regulator of ROS, apoptosis, and inflammation, the expression of KDM3A was also gradually increased with the extension of HG treatment time and peaked at 6 h, which was maintained for 48 h. Meanwhile, the expression of H3K9me2 was gradually declined and reached the minimum value at 24 h, which was maintained for 48 h (Figure 1E). Moreover, even if the glucose concentration returned to normal, KDM3A was still highly expressed while H3K9me2 remain expressed at a relatively low level (Figure 1F). As a result, we proposed the hypothesis that KDM3A might be a crucial mediator of HG-induced long-lasting cardiomyocytes dysfunction.

### Down-Regulation of KDM3A Could Repress While Up-Regulation of KDM3A Could Aggravate Continuous Reactive Oxygen Species Generation, Apoptosis and Inflammatory Responses in Cardiomyocytes Induced by High Glucose

**FIGURE 2:**
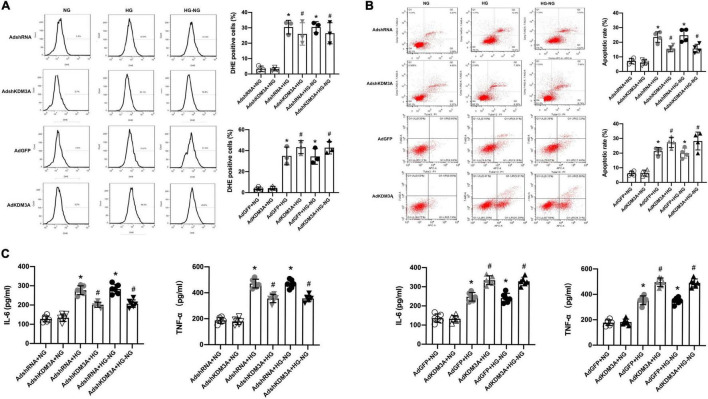
KDM3A knock-down could exacerbate while KDM3A over-expression could ameliorate continuous cardiomyocytes dysfunction induced by high glucose. **(A)** The ROS production was evaluated by DHE and flow cytometry, and the percentage of DHE positive cells in different groups were calculated (*n* = 3). **(B)** Flow cytometry was used to detect the apoptosis of cardiomyocytes, and the apoptotic rate of each group was quantitatively analyzed (*n* = 4). **(C)** To investigate the effects of KDM3A on inflammation under hyperglycemia stimulation, IL-6 and TNF-α in myocardial cells were measured using an ELISA kit (*n* = 4). *p < 0.05, as compared to the AdshRNA + NG or AdGFP + NG; ^#^*p* < 0.05, as compared to the AdshRNA + HG or AdshRNA + HG–NG, AdGFP + HG or AdGFP + HG–NG.

To verify this hypothesis, recombinant adenovirus vectors with KDM3A over-expression (AdKDM3A) and knock-down (AdshKDM3A) were successfully constructed (Supplementary Figures 1A,B), which could meet the needs of subsequent experiments. According to the results of flow cytometry, cardiomyocytes in HG and HG–NG groups generated more ROS than that in the NG group. The KDM3A knock-down significantly reduced ROS generation in both HG group and HG–NG group, whereas KDM3A over-expression remarkedly increased ROS generation in both HG group and HG–NG group. Moreover, no differences were found in the number of DHE positive cells between the HG and HG–NG groups after up-regulating or down-regulating KDM3A expression (Figure 2A). Additionally, KDM3A knock-down or over-expression have no perceptible effect on the apoptosis and inflammatory responses of cardiomyocytes when incubated in the NG medium. However, down-regulation of KDM3A obviously decreased HG and HG–NG triggered cell apoptosis and reduced the expression of the pro-inflammatory mediators IL-6 and TNF-α. However, KDM3A up-regulation exerted the opposite effects. Noteworthy, there were no statistical differences in apoptosis rates and inflammatory cytokines expression between HG and HG–NG groups regardless of up-regulation or down-regulation of KDM3A (Figures 2B,C). Data above indicated that KDM3A inhibition could regulate high glucose-induced long-term cardiomyocytes injury by modulating ROS generation, apoptosis, and inflammation.

### KDM3A Downregulation Suppresses the Expression and Transcriptional Activity of Nuclear Factor Kappa-B/P65 in High Glucose-Induced Cardiomyocytes Injury, While KDM3A Over-Expression Exert the Opposite Results

**FIGURE 3:**
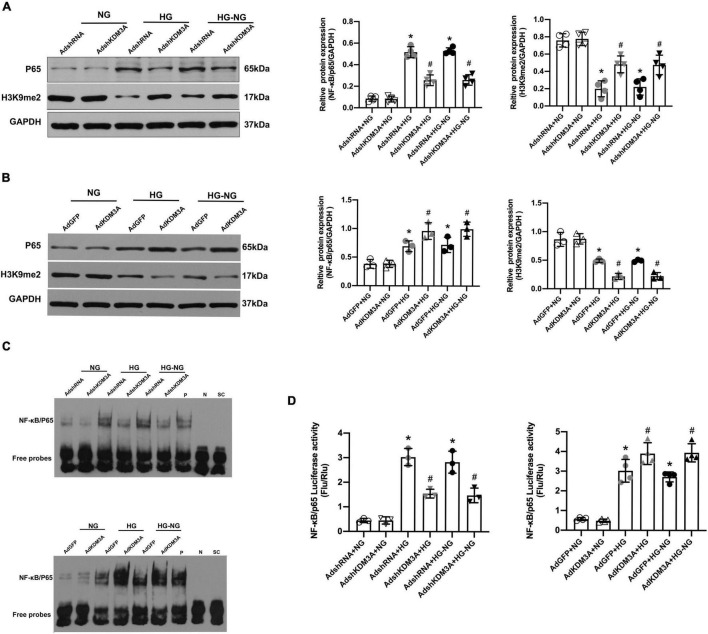
KDM3A modulates the expression and transcriptional activity of NF-κB/P65 in high glucose-induced persistent cardiomyocytes dysfunction. The primary cardiomyocytes were treated with HG 72 h or HG 24 h to NG 48 h (HG–NG) after the transfection with recombinant adenovirus vectors for 48 h, including AdshRNA, AdKDM3AshRNA, AdGFP, and Ad-KDM3A. **(A,B)** The protein levels of P65 and H3K9me2 were analyzed by western blot after down- or up-regulating the expression of KDM3A by adenovirus transfection (*n* = 4). **(C)** Furthermore, EMSA was used to assess the DNA binding activity of NF-κB in cardiomyocytes under different conditions. **(D)** To identify whether KDM3A directly targets NF-κB, the dual-luciferase reporter gene assay was conducted after down- or up-regulating the expression of KDM3A (*n* = 3). *p < 0.05, as compared to the AdshRNA + NG or AdGFP + NG; ^#^*p* < 0.05, as compared to the AdshRNA + HG or AdshRNA + HG–NG, AdGFP + HG or AdGFP + HG–NG.

In our previous study, we demonstrated that KDM3A/NF-κB/P65 signaling pathway was closely associated with ROS generation, apoptosis, and inflammation in VSMCs, suggesting that KDM3A may be a key upstream regulator of NF-κB/P65 (27). In addition, the investigators also confirmed the continuous activation of transcriptional activity of NF-κB/P65 were involved in the process of hyperglycemia-induced persistent myocardial injury (29). The evidence discussed here indicates a potential mechanistic relationship between KDM3A and NF-κB/P65 activation in HG-induced long-lasting cardiomyocytes injury. Thus, western blots were performed, and the results showed that the expression of P65 protein was increased after HG and HG–NG treatments, which was accompanied by an obvious reduction in the H3K9me2 expression. In addition, KDM3A knock-down inhibited NF-κB/P65 transcription and further up-regulated H3K9me2 expression under HG and HG–NG conditions (Figure 3A). However, KDM3A over-expression exerted the opposite effects (Figure 3B). Furthermore, the results of the Electrophoretic Mobility Shift Assay (EMSA) (Figure 3C) and the dual-luciferase reporter gene assay (Figure 3D) also showed that KDM3A down-regulation weakened the DNA binding ability and transcriptional activity of NF-κB/P65 in cardiomyocytes under both HG and HG–NG conditions. Nevertheless, KDM3A over-expression exerted the opposite effects. These results demonstrate that KDM3A may participate in the progression of HG-induced cardiomyocytes injury by regulating the expression and transcriptional activity of NF-κB/P65.

### Hyperglycemia-Induced Myocardial Injury and Cardiac Dysfunction Persisted Even After Glucose Levels Were Normalized, Accompanied by the Over-Expression of KDM3A

In view of the results obtained *in vitro*, we would like to further validate these conclusions *in vivo*. The results of fasting blood glucose monitoring showed that blood glucose in the DM and DM + GC groups were significantly increased from week 4 to week 12 and maintained at a relatively high level (blood glucose >13.9 mmol/L). However, after administration with insulin from week 12, the blood glucose level in the DM + GC group returned to normal (Supplementary Figure 2A). The above results indicate that the DM rat model was successfully established, and insulin treatment obviously controls the blood glucose level.

**FIGURE 4:**
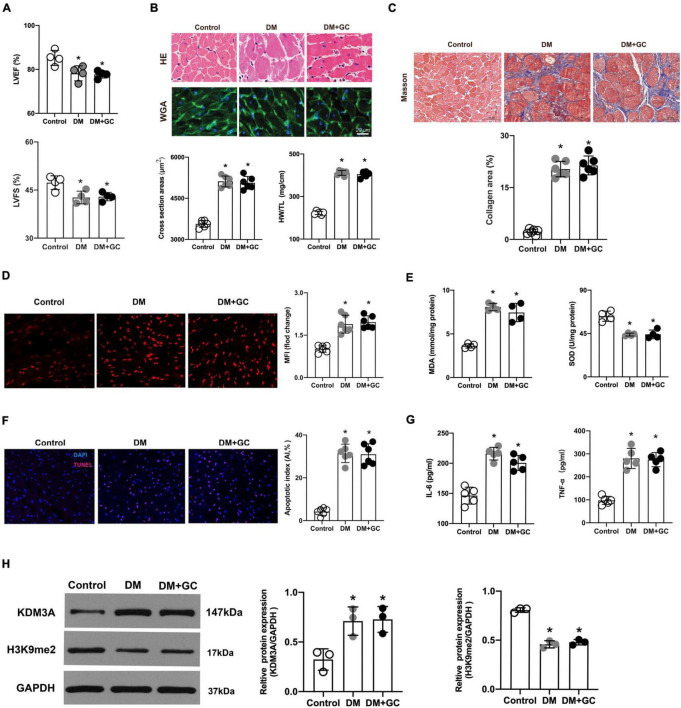
Hyperglycemia-induced cardiac dysfunction and myocardial injury persisted even after glucose levels were normalized, accompanied by the over expression of KDM3A. **(A)** The changes in cardiac function, including left ventricular ejection fraction (LVEF) and left ventricular short-axis fractional shortening (LVFS) were monitored by echocardiography in rats (*n* = 4). **(B)** The gross view of hearts, H&E and WGA staining of myocardium were performed. The cross-sectional area of myocardial cells and the ratio of heart weight to tibial length (HW/TL) in each group were calculated to evaluate cardiac hypertrophy (*n* = 5). **(C)** The degree of cardiac fibrosis was tested by Masson staining. The collagen area of each group was analyzed (*n* = 6). **(D)** The ROS production in rat myocardium was detected by DHE staining and the mean fluorescence intensity were analyzed (*n* = 5). **(E)** MDA content and SOD activity were tested to assess ROS generation indirectly (*n* = 4). **(F)** The apoptosis of myocardial cells was further estimated by TUNEL staining (*n* = 6). **(G)** The IL-6 and TNF-α content were measured using the ELISA method (*n* = 5). **(H)** The expression of KDM3A and H3K9me2 in normal rats (Control), diabetic rats (DM) and diabetic rats with glucose control by insulin (DM + GC) were also measured by western blot (*n* = 3). *p < 0.05, as compared to the Control group.

Compared with rats in the control group, the LVEF and LVFS were markedly decreased in the DM group. However, intensive blood glucose control exerted no evident improvement on cardiac function as compared with the DM group (Figure 4A). Meanwhile, diabetes-induced marked myocardial hypertrophy and fibrosis; however, the intensive blood glucose control neither reduced the cross-sectional area of cardiomyocytes and HW/TL ratio (Figure 4B) nor alleviate the degree of myocardial fibrosis (Figure 4C). As a result, it can be concluded that hyperglycemia exerts sustained impairment on cardiac function and lead to extensive myocardial injury and cardiac remodeling even after the blood glucose level was tightly controlled.

Consistent with the results from *in vitro*, DHE fluorescent probes and MDA/SOD detection results revealed that even if the glucose level returned to normal, the generation of ROS induced by hyperglycemia was still maintained at a high level (Figures 4D,E). Besides, the results of TUNEL fluorescence staining indicated that intensive blood glucose control did not significantly reduce cell apoptosis (Figure 4F). Additionally, the TNF-α and IL-6 levels increased in varying degrees in the DM group compared with that in the control group, while the intensive glucose lowering treatment did not evidently inhibit expression of TNF-α and IL-6 (Figure 4G). Evidence again indicated that persistent activation of ROS, apoptosis and inflammatory responses might be the key pathophysiological basis for hyperglycemia-induced sustained myocardial injury and cardiac dysfunction. Importantly, KDM3A expression was significantly increased accompanied by an obvious decrease in H3K9me2 expression in DM rats (Figure 4H), however, no statistical difference was observed in KDM3A or H3K9me2 expression between the DM and DM + GC groups.

### KDM3A-KO Could Alleviate Hyperglycemia-Induced Persistent Myocardial Injury and Cardiac Dysfunction *in vivo*

**FIGURE 5:**
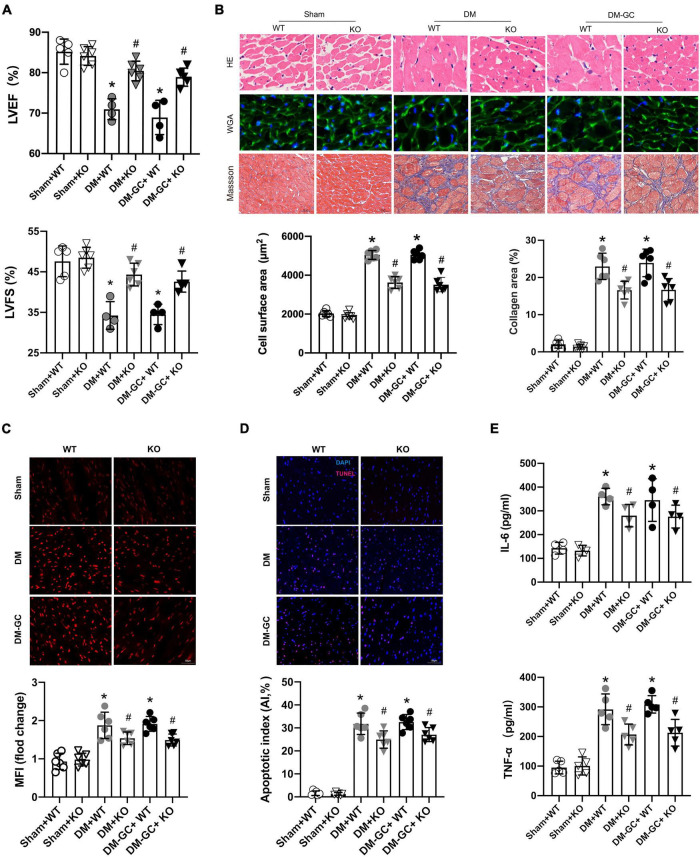
KDM3A knock-out could alleviate hyperglycemia-induced persistent myocardial injury and cardiac dysfunction *in vivo*. **(A)** The cardiac function was evaluated by echocardiography including LVEF and LVFS. **(B)** The degree of myocardial hypertrophy and fibrosis were detected by H&E (X400), WGA (X400), and Masson staining (C, X200). The quantitative analysis was conducted according to the cross-sectional area of myocardial cells and collagen area (*n* = 6). Furthermore, the ROS generation **(C)**, apoptosis in the myocardium **(D)** were estimated by DHE and TUNEL staining, respectively (*n* = 5). **(E)** The inflammation factors, including IL-6 and TNF-α in myocardium lysis, were measured using ELISA kits (*n* = 5). *p < 0.05, as compared to the Sham + WT or the Sham + KO group; ^#^*p* < 0.05, as compared to the DM + WT or the DM–GC + WT group.

We have attempted to construct cardiomyocyte-specific KDM3A knock-out and over-expression rats, which unfortunately resulted in embryonic death. Only rats with the global KDM3A knock-out were successfully constructed and used in this study. According to the results of screening and sequencing identification, KDM3A global gene knock-out chimeric Founder rats were successfully constructed (Supplementary Figures 2B–E). The KDM3A-KO did not affect cardiac function in the basal state when compared with the WT group. However, after the DM model was established, rats in the DM–KO group exhibited higher LVEF and LVFS levels than that in the WT + DM group. Moreover, in the diabetic rats that received intensive GC therapy, the cardiac function of KDM3A-KO rats was also significantly improved compared to the WT rats (Figure 5A). Meanwhile, it was found from HE/WGA staining and Masson staining results that after DM and DM + GC treatment, the cross-sectional area and fibrosis degree of myocardial cells in the WT group and KO group were prominently increased compared with those in the sham group. Consistent with the echocardiography results, KDM3A-KO obviously relieved myocardial hypertrophy and fibrosis compared with the WT rats after DM and DM + GC treatments (Figure 5B).

Therefore, KDM3A knock-out alleviates long-term myocardial dysfunction and hypertrophy as well as mitigates fibrosis caused by hyperglycemia in the diabetic myocardium.

### KDM3A-KO Could Suppress Hyperglycemia-Induced Continuous Activation of Reactive Oxygen Species Generation, Apoptosis and Inflammatory Responses *in vivo*

Consistent with the *in vitro* results, data of DHE staining demonstrated that after DM and DM + GC treatment, the accumulation of ROS was significantly increased in the WT and KDM3A-KO diabetic rats compared with that in the Sham group. However, ROS generation was significantly reduced in KDM3A-KO rats compared to WT rats in either the DM group or the DM-GC group (Figure 5C). In accordance with the ROS results, the TUNEL staining and IL-6/TNF-α results also showed that KDM3A knock-out evidently alleviated sustained apoptosis and inflammatory responses induced by hyperglycemia (Figures 5D,E).

### KDM3A-KO Inhibited the Expression of Nuclear Factor Kappa-B/P65 and Its Transcriptional Activity *in vivo*

**FIGURE 6:**
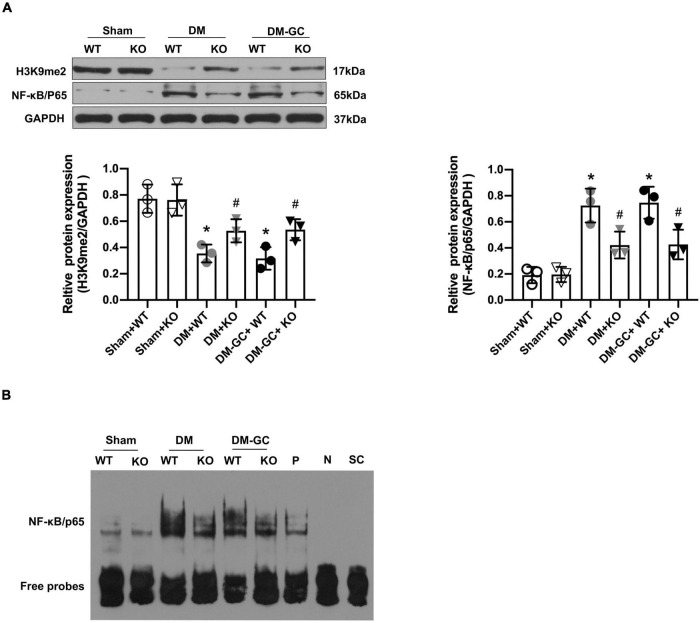
KDM3A regulates the expression and DNA binding ability of NF-κB/P65 in diabetic myocardium. **(A)** To explore whether KDM3A participates in regulating NF-κB/P65 under diabetic conditions, the proteins of P65 and H3K9me2 were detected by western blot (*n* = 3). **(B)** The DNA binding ability of NF-κB was assessed by the EMSA method. *p < 0.05, as compared to the Sham + WT or the Sham + KO group; ^#^*p* < 0.05, as compared to the DM + WT or the DM–GC + WT group.

The results of the western blot assay showed that the expression of P65 protein was significantly increased in both WT DM rats that didn’t receive intensive GC treatment and WT DM rats that received intensive GC treatment, accompanied by an obvious reduction in the H3K9me2 expression (Figure 6A). Moreover, the P65 protein level was significantly decreased and H3K9me2 protein expression was markedly increased in DM and DM + GC group after KDM3A knock-out (Figure 6A). Interestingly, there was no significant difference in P65 protein expression in both KDM3A-KO and WT rats between the DM and DM + GC groups. EMSA also demonstrated that the P65 DNA binding activity in the myocardium of DM rats continued to be increased even after being administrated with insulin, however, P65 DNA binding activity was remarkably declined after KDM3A knock-out in the DM and DM + GC groups (Figure 6B). The above *in vivo* results revealed the protective efficiency of KDM3A knock-out against hyperglycemia-induced persistent myocardial injury and cardiac dysfunction might be associated with the inhibition of the expression and transcriptional activity of NF-κB/P65.

### KDM3A Regulates High Glucose-Induced Persistent Cardiomyocytes Injury in an Nuclear Factor Kappa-B/P65-Dependent Manner

**FIGURE 7:**
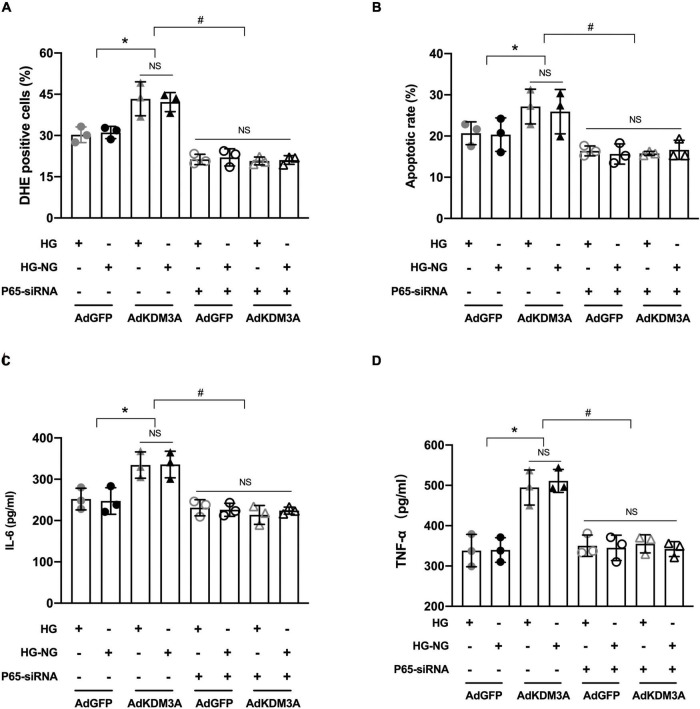
KDM3A regulates high glucose-induced cardiomyocytes dysfunction in an NF-κB/P65 dependent manner. To further investigate whether KDM3A affects hyperglycemia-induced impairment in diabetic myocardium through the P65/NF-κB dependent pathway, primary cardiomyocytes infected by AdKDM3A were also transfected with P65 siRNA plasmid, which was inhibiting P65 expression. Subsequently, these cells were given HG or HG–NG treatment, respectively. The ROS production **(A)**, apoptosis **(B)**, and inflammatory factors **(C,D)** were evaluated. *p < 0.05, as compared to the AdGFP + HG or AdGFP + HG–NG; *n* = 3, ^#^*p* < 0.05, as compared to the AdKDM3A + HG or AdKDM3A + HG–NG. Here, NS means no significant difference.

To further investigate whether NF-κB/P65 is the specific target for KDM3A regulating HG-induced persistent cardiomyocytes injury. The rescue experiments were performed to investigate whether P65 inhibition could block the biological function of KDM3A. Primary cardiomyocytes were infected with AdKDM3A or/and transfected with a P65-siRNA plasmid (P65 expression was inhibited). As shown in Figures 7A,B, P65 knock-down evidently alleviated KDM3A overexpression-induced excessive ROS production and apoptosis both in HG and HG–NG groups. Besides, P65 knock-down significantly ameliorated KDM3A over-expression-induced inflammatory reaction both in HG and HG–NG groups as well (Figures 7C,D). Data above demonstrated that P65 inhibition could partially contract KDM3A over-expression-induced persistent cardiomyocytes injury in the condition of HG.

## Discussion

**FIGURE 8:**
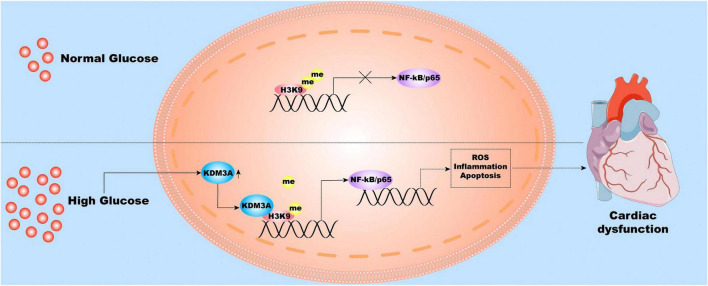
The molecular and cellular mechanisms underlying hyperglycemia-induced persistent myocardial injury and cardiac dysfunction. Hyperglycemia stimulation induces the over-expression of KDM3A; therefore, the KDM3A binds to the promoter region of the NF-κB/P65 gene and reduces the level of H3K9me2, thereby continuously promoting NF-κB/P65 transcription. As a result, NF-κB/P65 further results in long-lasting ROS accumulation, inflammatory reaction, apoptosis, and cardiac dysfunction.

In this study, our experiment once again demonstrated that the diabetic myocardium suffered a hyperglycemia-mediated injury, which was mainly characterized by extensive and long-lasting deterioration of cardiac structure and function, accompanied by persistent activation of ROS, inflammatory reaction, and apoptosis. Mechanically, KDM3A-NF-κB/P65 axis may function as a decisive signaling pathway in this pathological condition (Figure 8). Several lines of evidence support our conclusions, which are listed as follows: (1) The expression of KDM3A and NF-κB/P65 were significantly up-regulated in diabetes model and intensive GC model both *in vitro* and *in vivo*; (2) Persistent epigenetic signatures, namely histone hypomethylation, were observed even after intensive GC; (3) The KDM3A deletion down-regulated NF-κB/P65 expression, ameliorated hyperglycemia-induced myocardial injury, alleviated oxidative stress, inflammation, and apoptosis in both diabetes and intensive GC models. However, KDM3A upregulation exerted the opposite effect; (4) Moreover, NF-κB/P65 inhibition using siRNA could reverse the adverse effects caused by KDM3A over-expression. Therefore, our results clearly demonstrated that KDM3A contribute to increased chromatin accessibility and persistent NF-κB/P65 upregulation in the diabetic heart. KDM3A-NF-κB/P65 signaling pathway is expected to be a new therapeutic target for relieving hyperglycemia-induced persistent myocardial injury and cardiac dysfunction.

Although multiple elements such as hyperglycemia, insulin resistance, and abnormal lipid metabolism were involved in DCM, hyperglycemia was identified as the major pathogenic factor (30). Previous studies have confirmed that even if elevated blood glucose does not meet the diagnostic criteria for diabetes, hyperglycemia is still regarded as an independent risk factor for coronary heart disease (CHD) morbidity and mortality (31). Besides, in a community-based study of subjects that without clinically evident CHD, higher glycated hemoglobin (HbA1c) is associated with elevated high-sensitivity cardiac troponin-T (hs-cTnT) among persons without clinically evident CHD, indicating that hyperglycemia contributes to myocardial injury beyond its effects on the development of clinical atherosclerotic coronary disease (17). Several potential mechanisms have been confirmed implicated in hyperglycemia-induced myocardial injury, especially oxidative stress, inflammation, and apoptosis (32–34). Hyperglycemia and glucotoxicity could induce a protein glycation reaction leading to increases in advanced glycation end products (AGEs), which are produced from non-enzymatic glycosylation of lipids, lipoproteins, and amino acids. The ROS has been perceived participated in all stages of the development of DCM and AGEs were identified as pivotal factors that trigger ROS cascade reaction in diabetic myocardial, they mediate the damage of mitochondrial structure and function, accelerate the accumulation of intracellular ROS, facilitate the release of oxygen free radicals as well as promote excessive cardiomyocyte apoptosis (9, 35). In addition, hyperglycemia not only induced excessive mitochondrial ROS production but also stimulate inflammatory reactions (36). Previous studies have indicated that chronic and persistent inflammation might be one of the pivotal reasons that hyperglycemia causes structural and functional alterations in the cardiac muscle (37). Mechanically, the unbalanced glycemic and fatty acid metabolism increase the expression of inflammatory cytokines and cell adhesion molecules in the diabetic myocardial tissue (13, 38). The crosstalk between the inflammation and oxidative stress signaling molecules could further influence each other. Moreover, apoptosis induced by hyperglycemia is considered one of the hallmarks of DCM and has been verified to be associated with increased oxidative stress and inflammation (39). However, the patients with DM are still vulnerable to related cardiovascular complications after intensive blood glucose control. Early large-scale clinical trials, such as ADVANCE, UKPDS and VADT indicated that intensive hypoglycemic therapy can hardly reduce the risk of cardiovascular events in these patients (14). Besides, several large sample size meta-analyses also demonstrated that the risk of congestive heart failure did not differ significantly between the intensive GC group and standard treatment group (40, 41). Noteworthy, although some new types of hypoglycemic drugs such as Empagliflozin and Dapagliflozin have been affirmed effectively in reducing hyperglycemia burden, their benefits on heart failure are more likely driven by other mechanisms including osmotic diuresis, adjust plasma volume, endoplasmic reticulum stress and sodium retention as well as modulate the cardio–renal axis and re-balance the neurohumoral system (15, 42). In fact, hyperglycemia-induced continuous activation of ROS, inflammation, and apoptosis was considered as the core pathophysiological mechanism that mediates target organ injury. Consistent with this view, *in vitro* and *in vivo* data from this study once again uncovered the existence of persistent ROS accumulation, augmented inflammatory cytokine (IL-6 and TNF-α) expression, and increased apoptosis ratio in diabetic myocardium after glucose level returned to normal. Noteworthy, drugs with antioxidant, anti-inflammatory and anti-apoptotic properties have been shown to be effective in the treatment of myocardial damage caused by high glucose (10, 43–45). Therefore, targeting oxidative stress, inflammatory and apoptosis pathways are critical for the development of new therapies in hyperglycemia-induced myocardial injury.

Epigenetic mechanisms regulate gene expression and function without alerting the underlying DNA sequence and mediate crosstalk between genes and the environment (19, 20). Continuous epigenetic changes may be responsible for the hyperglycemia-induced sustained injury. Increasing evidence indicates that DM- or HG-triggered histone methylation is a crucial feature for persistent and extensive hyperglycemia-mediated cardiovascular injury. For instance, the histone methyltransferase SUV39H1-H3K9me3 pathway is a key factor that triggers the HG-mediated injury in VSMCs (22, 46). The histone methyltransferase Set7-H3K4me1 pathway has been shown to participate in HG-mediated injury in aortic endothelial cells (47). Furthermore, dysregulation of methyltransferase DNMT3b causes CpG demethylation on p66*^Shc^* promoter, leading to its persistent transcription and secondary cardiac dysfunction (15). As a member of the JMJC domain-containing proteins that belong to the histone demethylases family, KDM3A is deemed a key regulator in cardiovascular disease. According to recent studies, increased KDM3A expression in myocardial cells has a positive association with ANP/BNP transcription and participates in pressure-induced myocardial hypertrophy by regulating H3K9me2 levels (48). Our recently published study also indicated KDM3A take an active part in myocardial infarction-induced maladaptive remodeling by regulating inflammation and apoptosis (25). Interestingly, it has been shown that changes in KDM3A-mediated H3K9me2 expression are closely correlated with the left ventricular remodeling in patients with end-stage DCM (49). Besides. recent research also revealed that the KDM3A could change the H3K9me2 level of the target gene in the promoter region and participate in diabetic vessels injury *via* modulating oxidative stress, inflammatory and apoptosis (26). In the light of these considerations, we speculated that KDM3A may be a central joint between methylated modification and the regulation of oxidative stress, inflammatory reaction, apoptosis. In this study, we found that hyperglycemia induces continuous ROS generation, inflammatory responses, and apoptosis, accompanied by the over-expression of KDM3A and down-regulation of H3K9me2 even after glucose levels were normalized. However, KDM3A knock-down could mitigate these adverse reactions, ameliorate myocardial injury, and upregulate the expression of H3K9me2. The specificity of the above reaction further implies that KDM3A-mediated H3K9me2 changes may be a vital molecular mechanism for modulating hyperglycemia-induced injury.

As a classical transcription factor, NF-κB/P65 has been confirmed involved in multiple cellular pathophysiological processes. Previous studies indicated that NF-κB signaling pathway was activated in DCM *in vivo* and in cardiomyocytes induced by HG condition *in vitro* (32, 50). Mechanically, NF-κB/P65 activation may act in DCM *via* canonical and non-canonical pathways by controlling a different set of genes and being involved in related processes such as oxidative stress, inflammation, fibrosis, hypertrophy, and apoptosis (10, 51, 52). Additionally, there are also reports on the role of histone methylation-related NF-κB/P65 epigenetic regulation in hyperglycemia-mediated injury in the diabetic target organs. For example, SET7 mediates the recruitment of H3K4me1 to the promoter region of the P65 gene, thus giving rise to a continuous increase of NF-κB/P65 and exacerbating short exposure to high glucose-induced persist aortic endothelial cells injury (47). Moreover, our published research provided solid evidence that KDM3A functions as an upstream regulator of NF-κB/P65 and is implicated in hyperinsulinemia-caused VSMCs injury by aggravating inflammation, apoptosis, and ROS (27). The result of this study is in agreement with previous studies demonstrating enhanced activation of NF-κB pathway in both hyperglycemia myocardial injury model and diabetic intensive GC model. Also, the subsequent rescue experiment uncovered inhibition of NF-κB/P65 could block KDM3A-induced excessive ROS production, apoptosis, inflammatory reaction and myocardia injury in both HG and HG–NG groups. After considering these results, we concluded that KDM3A facilitates the expression and transcriptional activity of NF-κB/P65, thus leading to persistent myocardial injury in the context of hyperglycemia.

In summary, the diabetic myocardium suffers from a hyperglycemia-induced sustained injury, even after the glucose level returned to normal. Functional experiments showed that KDM3A inhibition could efficaciously reduce ROS production, decrease the inflammatory reaction, and ameliorate myocardial apoptosis, thereby improving cardiac function as well as inhibiting maladaptive myocardial remodeling. Mechanistically, we demonstrated that KDM3A mediates hyperglycemia-induced injury in the diabetic myocardium mainly through enhancing the expression and transcriptional activity of NF-κB/P65. In general, this study provided new insight into the regulatory mechanism of long-term diabetic myocardial damage and the KDM3A-NF-κB/P65 pathway might be a promising intervention target. However, there are several limitations in this study. Firstly, we mainly focus on the hyperglycemia-induced continuous injury this time, many other risk factors such as hyperinsulinemia and hyperlipemia were also involved in diabetic myocardial injury. The role of these factors remains to be explored. In addition, due to fetal death, we are unable to obtain KDM3A myocardial specific knock-out and over-expression rats. Whether other types of cells other than cardiomyocytes are involved in KDM3A-mediated diabetic myocardial injury *in vivo* are remain unknown. Moreover, transcription factors other than NF-κB/P65 may also serve as the downstream regulator of KDM3A and participate in the pathological process. As a result, further studies are still needed to address these problems.

## Data Availability Statement

The original contributions presented in the study are included in the article/Supplementary Material, further inquiries can be directed to the corresponding author.

## Ethics Statement

The animal study was reviewed and approved by Animal Care and Use Committee of Wuhan University.

## Author Contributions

BZ and JZ performed the study, analyzed, and interpreted the data, and wrote the manuscript. BZ, JC, GL, and XL made substantial contributions to the acquisition of data and manuscript revision. JC, BZ, and JZ performed experimental hypotheses and participated in the experimental design and data revision. JC, BZ, XG, and JZ performed the interpretation of data and manuscript drafting. All authors read and approved the final manuscript.

## Conflict of Interest

The authors declare that the research was conducted in the absence of any commercial or financial relationships that could be construed as a potential conflict of interest.

## Publisher’s Note

All claims expressed in this article are solely those of the authors and do not necessarily represent those of their affiliated organizations, or those of the publisher, the editors and the reviewers. Any product that may be evaluated in this article, or claim that may be made by its manufacturer, is not guaranteed or endorsed by the publisher.
