# Corrigendum: KDM3A inhibition ameliorates hyperglycemia-mediated myocardial injury by epigenetic modulation of nuclear factor kappa-B/P65

**DOI:** 10.3389/fcvm.2023.1231599

**Published:** 2023-06-20

**Authors:** Bofang Zhang, Jing Zhang, Gen Liu, Xin Guo, Xiaopei Liu, Jing Chen

**Affiliations:** ^1^ Department of Cardiology, Renmin Hospital of Wuhan University, Hubei Key Laboratory of Cardiology, Cardiovascular Research Institute, Wuhan University, Wuhan, China; ^2^ Department of Cardiology, the First College of Clinical Medical Science, Yichang Central People's Hospital, China Three Gorges University, Yichang, China

**Keywords:** hyperglycemia, cardiac dysfunction, epigenetic regulation, KDM3A, NF-κB/p65

A Corrigendum on KDM3A Inhibition ameliorates hyperglycemia-mediated myocardial injury by epigenetic modulation of nuclear factor kappa-B/P65 By Zhang B, Zhang J, Liu G, Guo X, Liu X and Chen J. (2022). Front. Cardiovasc. Med. 9:870999. doi: 10.3389/fcvm.2022.870999

**Error in Figure**

In the published article, there was an error in Figure 4 as published. Due to errors whilst copying and assembling pictures, we put the wrong images in Figures 4D,F. The corrected Figure 4 and its caption—“Hyperglycemia-induced cardiac dysfunction and myocardial injury persisted even after glucose levels were normalized, accompanied by the overexpression of KDM3A”—appear below.

**Figure 4:**
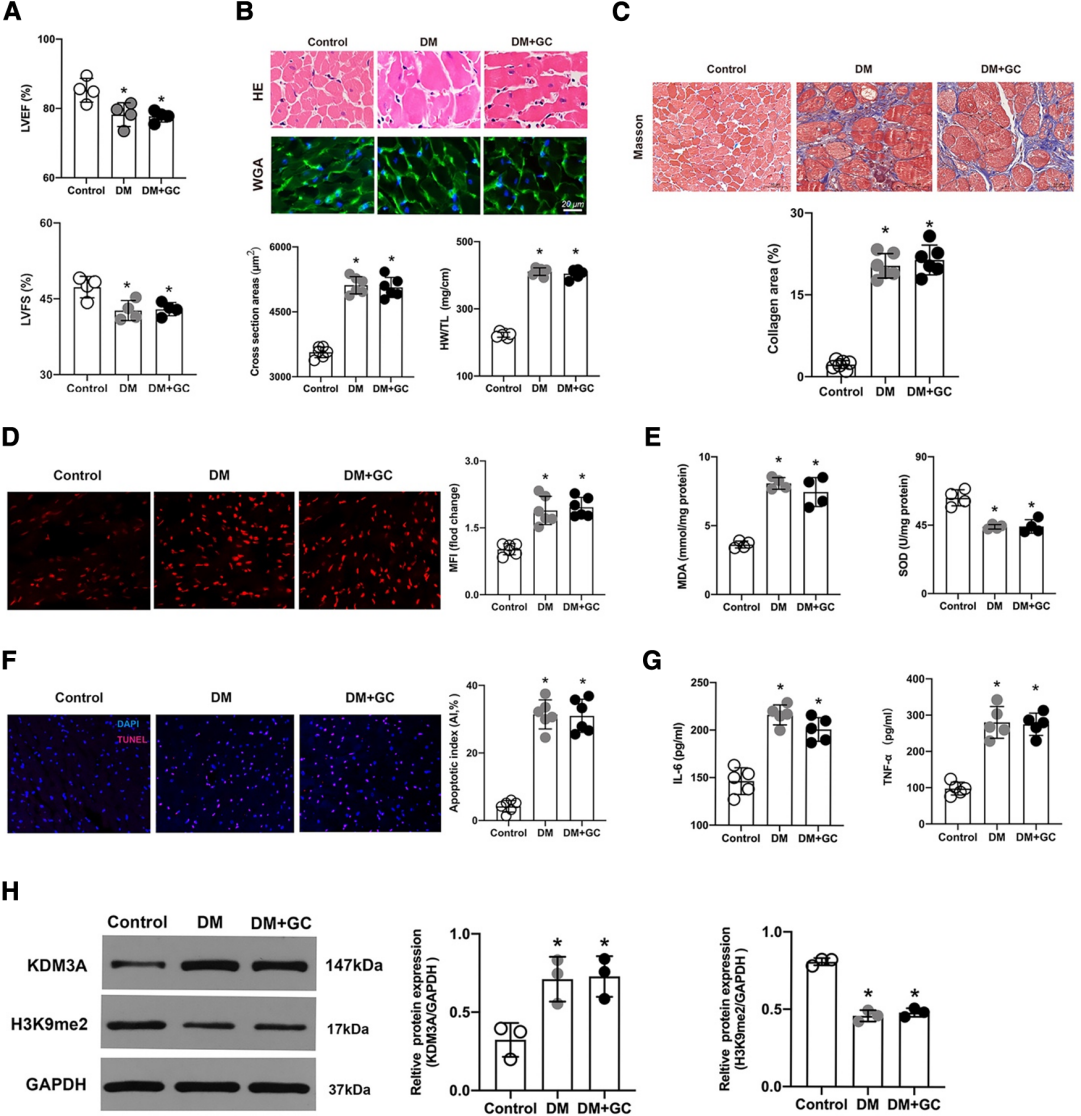
Hyperglycemia-induced cardiac dysfunction and myocardial injury persisted even after glucose levels were normalized, accompanied by the overexpression of KDM3A. (**A**). The changes in cardiac function, including left ventricular ejection fraction (LVEF) and left ventricular short-axis fractional shortening (LVFS) were monitored by echocardiography in rats (*n* = 4). (**B**). The gross view of hearts, H&E and WGA staining of myocardium were performed. The cross-sectional area of myocardial cells and the ratio of heart weight to tibial length (HW/TL) in each group were calculated to evaluate cardiac hypertrophy (*n* = 5). (**C**). The degree of cardiac fibrosis was tested by Masson staining. The collagen area of each group was analyzed (*n* = 6). (**D**). The ROS production in rat myocardium was detected by DHE staining and the mean fluorescence intensity were analyzed (*n* = 5). (**E**) MDA content and SOD activity were tested to assess ROS generation indirectly (*n* = 4). (**F**). The apoptosis of myocardial cells was further estimated by TUNEL staining (*n* = 6). (**G**). The IL-6 and TNF-α content were measured using the ELISA method (*n* = 5). (**H**) The expression of KDM3A and H3K9me2 in normal rats (Control), diabetic rats (DM) and diabetic rats with glucose control by insulin (DM + GC) were also measured by western blot (*n* = 3). *p < 0.05, as compared to the Control group.

The authors apologize for this error and state that this does not change the scientific conclusions of the article in any way. The original article has been updated.

