# Supplementary material for: KDM3A Inhibition Ameliorates Hyperglycemia-Mediated Myocardial Injury by Epigenetic Modulation of Nuclear Factor Kappa-B/P65
Source: Front Cardiovasc Med. 2022 Apr 29;9:870999. doi: 10.3389/fcvm.2022.870999 (PMC9106140; doi:10.3389/fcvm.2022.870999)

Supplementary figures

Supplementary Figure 1.

cardiomyocyte infected with AdshKDM3A markedly decrease the expression of KDM3A while AdKDM3A evidently increased the expression of KDM3A.

A, Protein level of KDM3A in NRCMs infected with AdshKDM3A. (n=3, **P*<0.05 vs. AdshRNA). B. Protein level of KDM3A in NRCMs infected with AdKDM3A (n=3, **P*<0.05 vs. AdGFP).

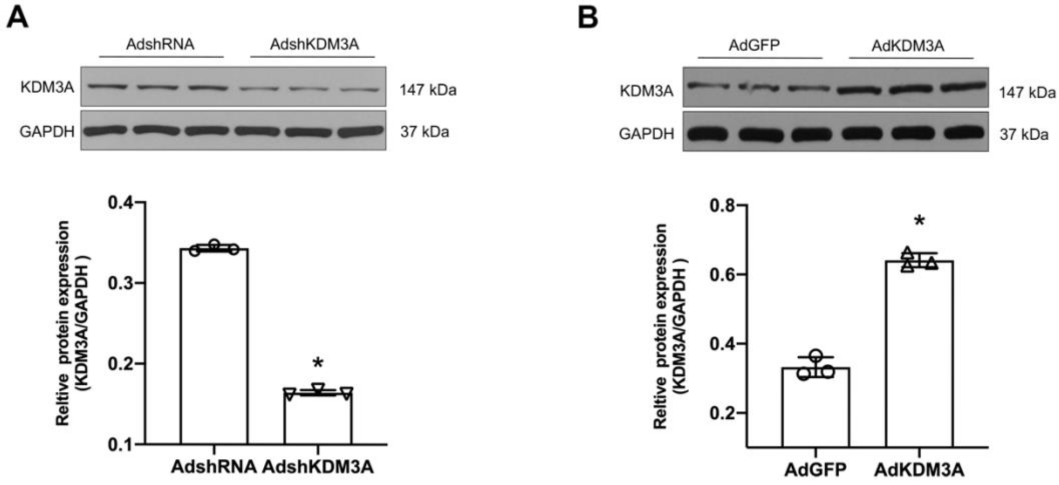

Supplementary Figure 2.

The establishment of T2DM model and T2DM intensive glycaemic control model. A CRISPR/Cas9 genome-editing technology was operated to generate KDM3A-KO (*$kdm3a^{-/-}$*) rat.

Rats were given high-fat diet (HDF) for 4 weeks followed with a low dose of STZ, to establish T2DM models, which were taken as the DM group. Some T2DM rats were subcutaneously injected with insulin (3 U/d) on 12 weeks continued for another 8 weeks, which were served as the DM+GC group. Rats in the DM and DM+GC group were given HFD for 20 weeks. While rats in the Control group were fed with normal diet for 20 weeks. The fasting blood glucose was continuously monitored every two weeks (A, n=6). One single guide RNA (sgRNA) flanked exon 5 of KDM3A gene in rat was designed and created (B). PCR gel electrophoresis show KDM3A products on wild type (*$kdm3a^{+/+}$*), heterozygote *$kdm3a^{+/-}$* and homozygous *$kdm3a^{-/-}$* hearts (C). The sequencing chromatograms of homozygous mutants and *$kdm3a^{+/+}$* heart revealed a 14 bases absence (D). The expression of KDM3A in the myocardium of WT rat and KDM3A-KO rat (E).

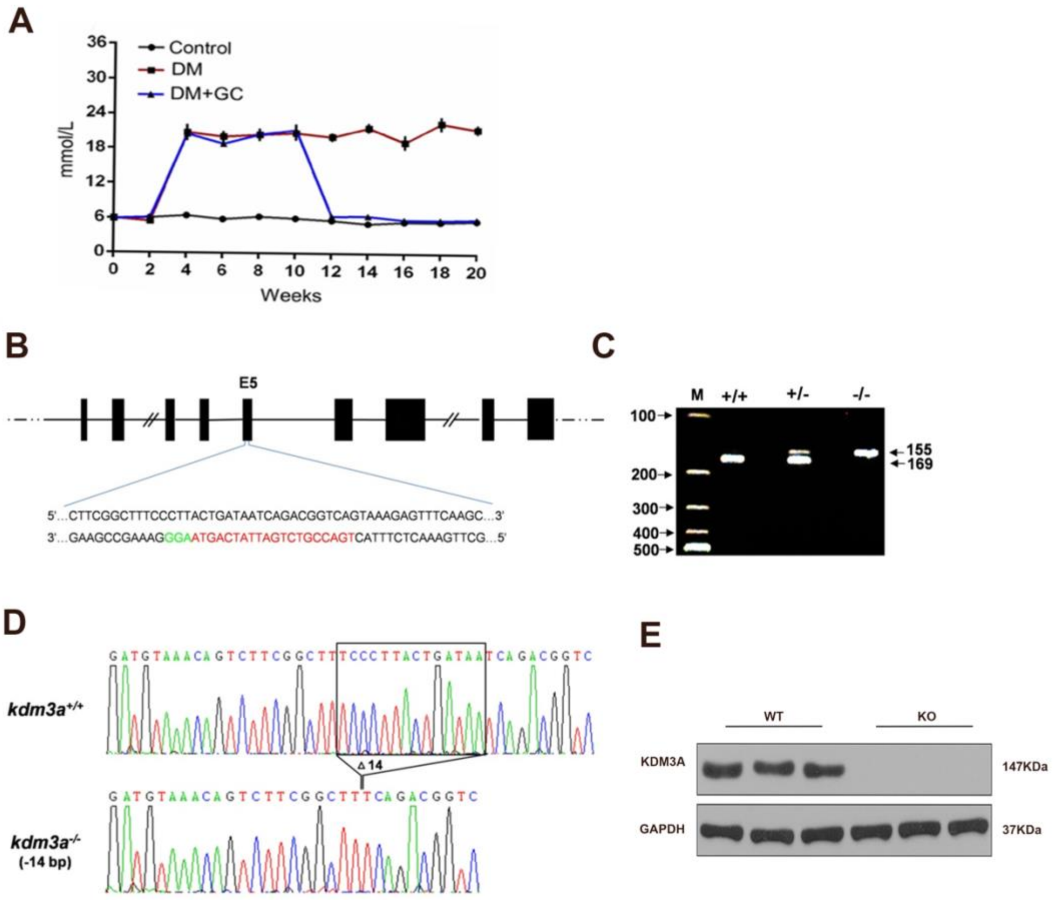

Supplement: Supplementary file 1 [file Data_Sheet_1.PDF]
