# Supplementary figures and images for: KDM3A Inhibition Ameliorates Hyperglycemia-Mediated Myocardial Injury by Epigenetic Modulation of Nuclear Factor Kappa-B/P65
Source: Front Cardiovasc Med. 2022 Apr 29;9:870999. doi: 10.3389/fcvm.2022.870999 (PMC9106140; doi:10.3389/fcvm.2022.870999)

Supplementary Figures

Figure 1.

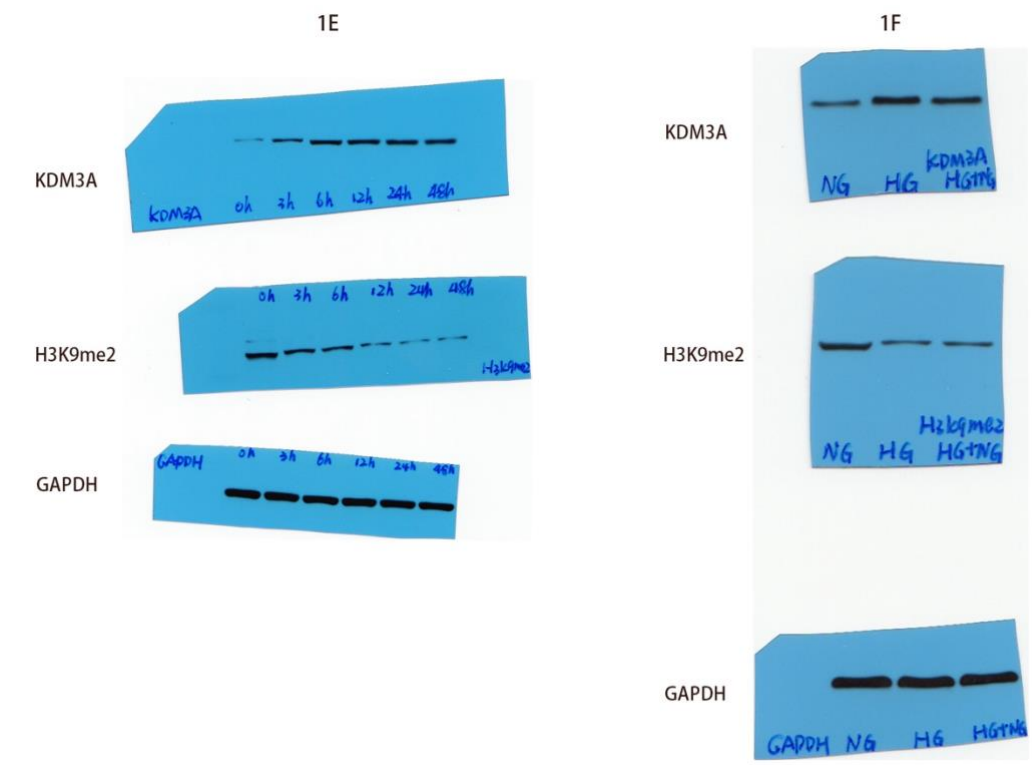

Figure 2.

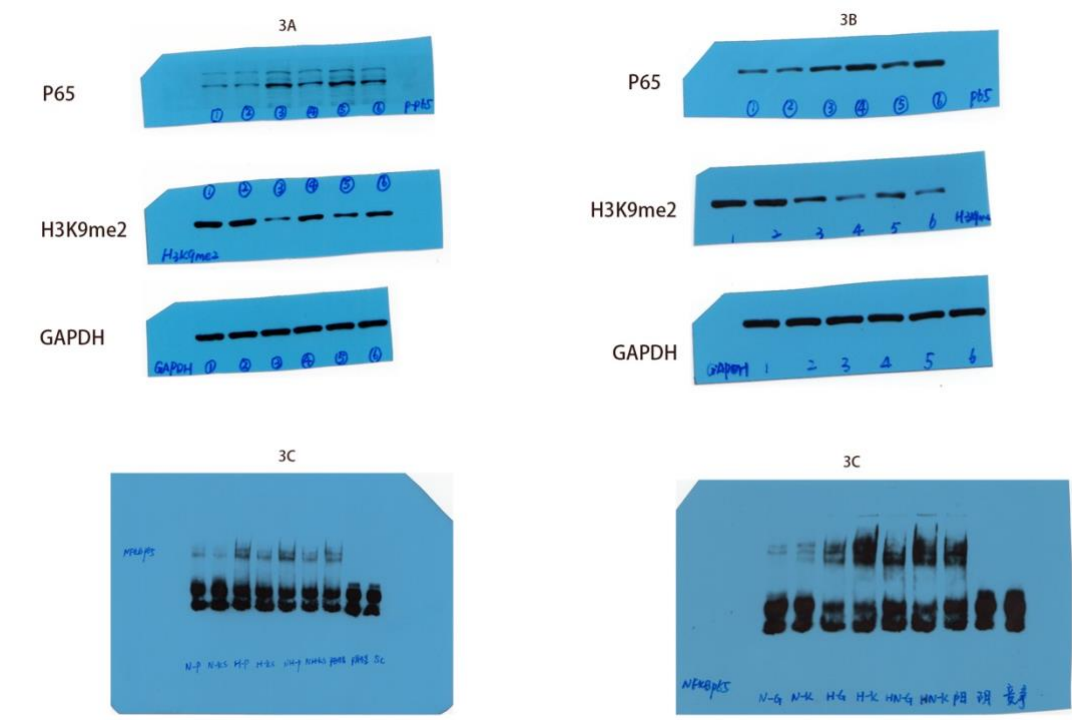

Figure 3.

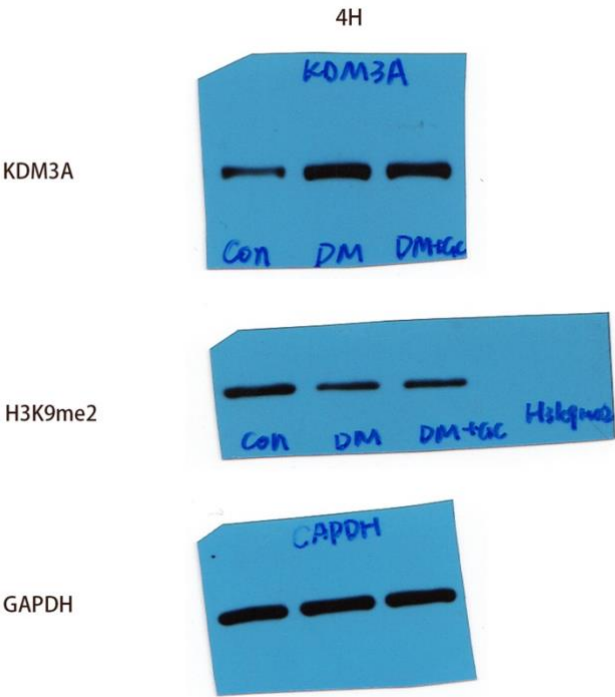

Figure 4.

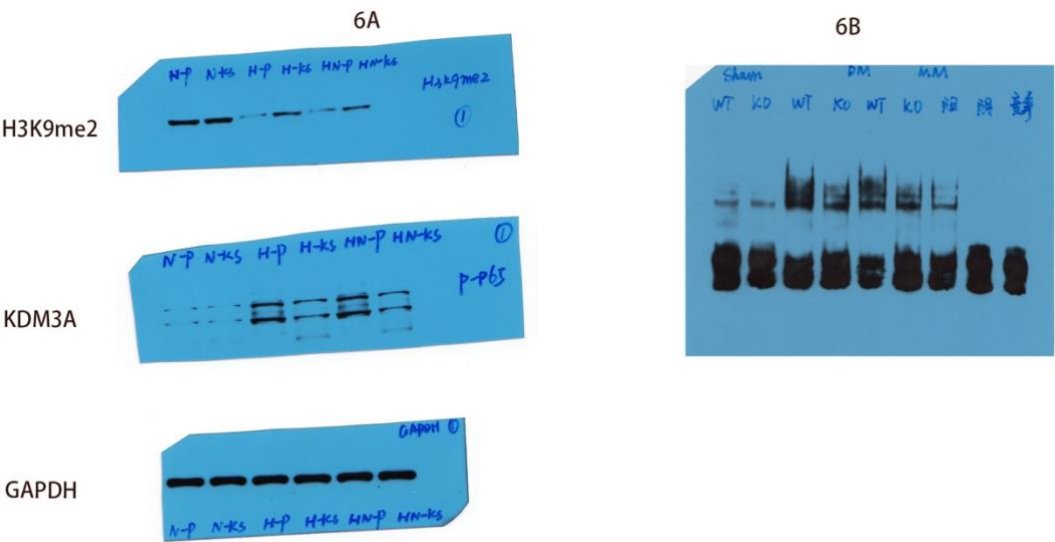

Figure 5.

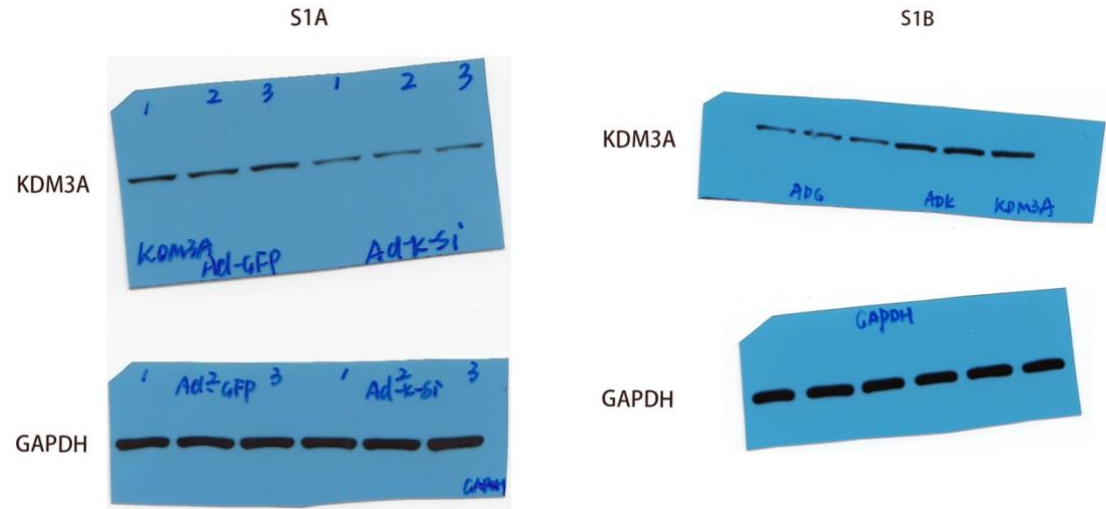

Figure 6.

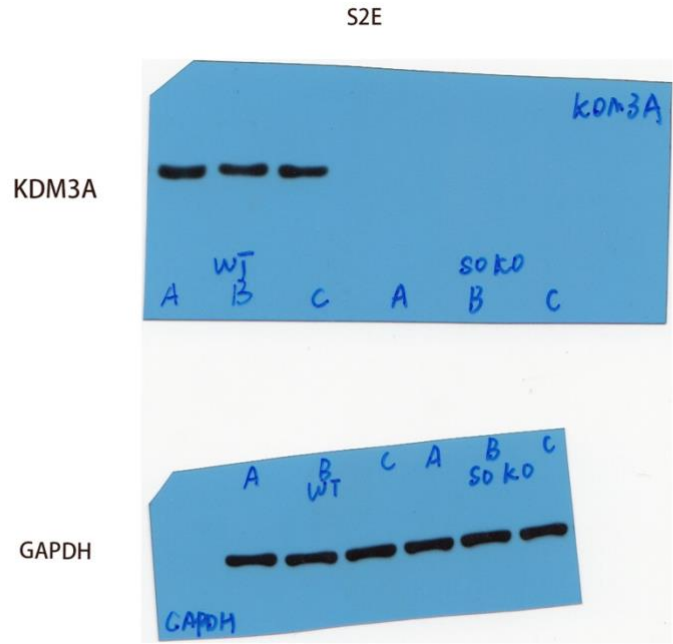

Supplement: Supplementary file 2 [file Data_Sheet_2.PDF]
